# 3D Analysis of Human Movement, Sport, and Health Promotion

**DOI:** 10.3390/jfmk8040157

**Published:** 2023-11-07

**Authors:** Luca Petrigna, Giuseppe Musumeci

**Affiliations:** Department of Biomedical and Biotechnological Sciences, Section of Anatomy, Histology and Movement Science, School of Medicine, University of Catania, Via S. Sofia 97, 95123 Catania, Italy; giuseppe.musumeci@unict.it

This Special Issue, “3D Analysis of Human Movement, Sport, and Health Promotion”, aimed to collect studies that assessed motor functions and alterations. The idea was to focus attention on objective and quantitative evaluation methods in both sport and, primarily, health promotion. According to the World Health Organization, health is a “complete physical, mental and social well-being, and not merely the absence of disease or infirmity” [1]. It is consequently important to think about well-designed health interventions in this way, to reduce the risks of correlated health status problems and reduce costs in healthcare systems [2,3]. On the one hand, the education of the population is fundamental for better knowledge on healthy habits; on the other hand, it is important to perform regular screening to evaluate health status [4,5]. 

It is possible to evaluate health status with laboratory tests (which usually present higher reliability and validity) and field tests (which are cheaper and easier to administer) [6]. Usually, field tests are more ecologically valid, making them a good solution for population-based studies [7]. Both solutions have to be investigated to achieve high-quality evaluations, as well as tests that can be performed involving more people in less time. One aspect that laboratory and field evaluations should have in common is the scientific quality of the protocol. Recently, the concept of the standard operating procedure in physical fitness evaluation has been proposed [8]. The idea of this concept is a detailed step-by-step description of the protocol to increase the quality of the research, allow the repetition of the protocol, and compare the data [8]. It is a concept adopted in other disciplines such as in the fields of medicine and engineering, where errors are not allowed [8]; consequently, it should also be suitable for the field of health evaluation.

The importance of evaluation with three-dimensional (3D) analysis is that it allows a vision of the human body on the three planes to be obtained, increasing the possibility of better understanding all aspects that could bring functional alterations [9]. The idea behind 3D testing is that kinematic analysis, especially if performed in 3D, helps in the evaluation of the quality of movements [10]. This is extremely helpful in the rehabilitation setting, as the study of the human kinematics for movement quantification is fundamental for evaluating improvements in patients, such as after a stroke [11,12], in scoliosis evaluation [13], or for digital postural analysis [14].

The articles included in this Special Issue address the 3D evaluation of human movement in different populations, the practical application of postural analysis, and possible future directions of research in this field. Different methods could be adopted in 3D evaluation, such as multicamera systems, machine learning approaches, or specific software for computers and smartphones. One of the studies included in this Special Issue adopted four infrared 3D cameras using the SMART Integrated System [15]. Russo and colleagues, in their article [15], evaluated the impact of Nordic walking pole length on healthy participants. The authors of [15] adopted a technique that is considered the gold standard for dynamic evaluation, but the price of the instrument limits its use. An alternative method detected in the included studies in the present Special Issue for human 3D evaluation is photogrammetry. It is a radiation-free, easy, inexpensive, and rapid tool that can help in postural screening and repetitive controls. It was adopted in the study by Belli and colleagues, who suggest that this instrument works especially well in bending evaluation [16]. 

The above systems are not always accessible for all clinicians due to their cost and the skills required to use them, suggesting the use of more economical techniques such as the calculation of body angles from photographs or the use of goniometry may be more attainable [17]. Following this principle, the study in [18] adopted software that evaluated human posture after the positioning of markers on specific anatomical landmarks. The authors in [18] analyzed body posture accurately and the software adopted was Dartfish ProSuite 6 (Dartfigh, Fribourg, Switzerland). Another study [19] adopted a mobile app named Apecs (Apecs-AI Posture Evaluation and Correction System^®^. New Body Technologies SAS, Grenoble, France). This application demonstrated good reproducibility with trunk inclination, although axillae alignment was unreliable in all the planes. This evaluation is cheap, easy to adopt (the application indicates the anatomical points on which to place the markers), and feasible; consequently, it could be an alternative to more expensive devices.

Software recording with 3D systems sometimes requires the use of technology to analyze data. In the life sciences, there is an ever-increasing interest in machine learning [20]. This technology is based on deep learning and has multiple field applications; one that could be of interest for movement analysis is the identification of objects in images [21]. Machine learning tools are useful for analyzing 3D movement kinematics and they can help distinguish healthy from pathological behaviors [10]. Deep learning could be adapted to analyze behaviors with standardized methods proposed in the literature [22]. In one of the studies included in this Special Issue, a machine learning approach was adopted in the evaluation of climbing holding time [23]. The concept of the study could also be adopted in other studies using 3D data.

All the above aspects should be related with their practical applications. Using 3D analysis is not only useful for researchers but also for kinesiologists. Three-dimensional evaluation and analysis could be useful in a studio, in a sports setting, or in a rehabilitation setting, but could also be performed remotely. Indeed, new technologies, if integrated with proper applications and supported by a good internet connection, could allow for the monitoring of treatment, as was demonstrated in another study included in this Special Issue [18]. As a review suggests [24], everything proposed above could be moved to the metaverse, a place where these new technologies and evaluation techniques can be adopted to reach people everywhere and at every time.

A practical application proposed in another study is the evaluation of hand-standing from a posturographic point of view [25]. It is not a natural human position, but different sports and disciplines require this specific and not-so-often-studied posture. Consequently, the study provides feedback for future research on this topic. Future research could also consider the evaluation of the human posture by integrating the above-presented tests with other techniques such as a termocamera [14] or in dual-task conditions [26]. In this way, it is possible to increase the quality and accuracy of the evaluation.

This Special Issue seeks to provide an overview of instruments, research ideas, and also future applications of 3D analysis technology. In the near future, it will be interesting to make some of the above-presented instruments the gold standard or valid research tools that could complement much more expensive and complex instruments. Furthermore, researchers should start to think about adopting new markerless technologies or techniques to make research as objective, precise, and cheap as possible.

In conclusion, this editorial aimed to present an overview of the Special Issue “3D Analysis of Human Movement, Sport, and Health Promotion” and provide feedback for future studies on the same or similar topics.
